# Physical Activity, Bleedings and Quality of Life in Subjects with Haemophilia A without Inhibitors—A Multicenter, Observational Italian Study with a Wearable Device

**DOI:** 10.3390/jcm13113036

**Published:** 2024-05-22

**Authors:** Maria Elisa Mancuso, Chiara Biasoli, Renato Marino, Andrea Buzzi, Daniele Preti, Luigi Sannino, Rosaria Tempre, Sara Bendinelli, Elena Pompeo, Giacomo Siri, Giancarlo Castaman

**Affiliations:** 1 Center for Thrombosis and Hemorrhagic Diseases, IRCCS Humanitas Research Hospital and Humanitas University, 20089 Milan, Italy; mariaelisa_mancuso@libero.it; 2 Centro Emofilia, Unità Opertaiva Complessa Medicina Trasfusionale, Dipartimento Patologia, Clinica Ospedale M. Bufalini, CESENA, 47521 Cesena, Italy; chiara.biasoli@auslromagna.it; 3 Ospedale Policlinico, S.S.D. Centro Emofilia e Trombosi, Piazza Giulio Cesare, 70124 Bari, Italy; renato.marino@policlinico.ba.it; 4 Fondazione Paracelso, 20155 Milan, Italy; andrea.buzzi@fondazioneparacelso.it; 5 Federazione Associazioni Emofilici, 20155 Milan, Italy; daniele.preti@fedemo.it; 6 Roche Italia, Viale G. B. Stucchi, 110, 20900 Monza, Italy; luigi.sannino@roche.com (L.S.); rosaria.tempre@roche.com (R.T.); elena.pompeo@roche.com (E.P.); 7 Alira Health S.r.l., Via Dante 14, 20121 Milan, Italy; siri.giacomo03@gmail.com; 8 Center for Bleeding Disorders and Coagulation, Department of Oncology, Careggi University Hospital, 50134 Florence, Italy; giancarlo.castaman@unifi.it

**Keywords:** cohort studies, hemophilia A, factor VIII deficiency, congenital, activity, physical, quality of life, bleeding, pain, patient outcome

## Abstract

**Background:** This study aimed to gather data on physical activity (PA), bleeding, health-related quality of life, and health status, using a wearable device and an electronic patient-reported outcome (ePRO) app, in individuals with moderate or severe hemophilia A (HA) without inhibitors receiving treatment according to the clinical practice. **Methods:** This is a 12-month multicenter cohort study conducted in Italy. The primary outcomes included the description of PA by type and intensity, adherence to World Health Organization guidelines, bleeding, and health-related quality of life by EQ-5D questionnaire. PA data were collected continuously through a fitness tracker worn by the patient; all the other variables were collected through ePRO questionnaires. **Results:** Only 54 of the 103 enrolled subjects (52.4%) used their fitness tracker for the defined valid period; adolescents were the least compliant age group. PA was performed at low rates and intensity. Approximately 52% of the subjects had sedentary behavior. The mean EQ-5D values did not change over time. At least one bleeding was reported in 43.7% of the subjects, mostly with sedentary behavior. The PA in the 2 days preceding the bleeding was comparable to the one observed in the overall observational period. **Conclusions:** The systematic recording of data through a fitness tracker and ePRO app shows that subjects with HA without inhibitors have lower-than-expected PA and that they still experience issues related to bleeding.

## 1. Introduction

Subjects with congenital FVIII deficiency (hemophilia A—HA) [1,2] suffer from bleeding episodes, especially into the joints or muscles, that cause pain and can lead to chronic swelling, deformity, reduced mobility, and long-term joint damage [3]. The absence or functional deficiency of FVIII leads to bleeding tendency [3], reduced bone mineral density [4,5,6], and causes prolonged bleeding after trauma [3]. Prevention of bleeding through prophylaxis, versus the on-demand treatment of bleeding events, is considered the gold standard of treatment for severe HA [5] and nowadays it can be effectively achieved with FVIII replacement or non-replacement therapies.

Regular physical activity (PA) is recommended for subjects with HA to preserve function and rehabilitate joints and muscles after bleeding episodes [7]. Moreover, improving mobility and activity levels in this population are important goals that positively influence their quality of life (QoL) [7,8]. Scientific evidence and guidelines support the health-promoting value of PA, underlining its importance in subjects with HA across all ages [4,5]. In a recent study, PA levels were equivalent to controls in males aged 13 to 30 who were receiving prophylactic treatment for their HA. However, a significant portion of adolescents failed to perform the required amount of moderate-to-vigorous PA each week [9], even if they experienced a negligible amount of bleeding and had good joint status [10].

In adults with HA, limited prospective data have been collected about the impact of bleeding episodes on PA and the resulting activity limitations.

This study aimed to collect information about PA level, risk of bleeding, QoL, and health status in subjects with moderate or severe HA without inhibitors, treated according to routine clinical practice.

## 2. Materials and Methods

This was a multicenter, non-interventional, prospective study in which subjects with severe and moderate HA were observed for 12 months or up to 18 months if a switch to a different drug occurred during the study.

The study started on 14 January 2020 (first patient in) and was completed on 28 April 2022 (last patient last visit).

### 2.1. Rationale for Study Design

In subjects with HA without inhibitors, bleed control and prevention can be effectively achieved with prophylactic treatment [11,12]. However, there is little information on the impact of bleeding on PA, as well as on the risk of bleeding related to PA and quality of life in this setting [13]. Persons with HA without inhibitors and crippling disabilities have been considered an appropriate population for evaluating these outcomes.

The study was conducted to collect information on PA status, bleeds, HRQoL, and health status in patients with severe or moderate HA without inhibitors receiving standard-of-care treatment, during a period of 12–18 months of routine clinical management; thus, the observational approach has been the most appropriate.

### 2.2. Electronic Patient-Reported Outcomes with Bring-Your-Own-Device (BYOD)

Advances in technology have significantly increased electronic patient-reported outcomes (ePRO) data collection capabilities coons [4,6,14,15]. An approach that leverages patients’ own devices to enable the collection of self-report data (“Bring Your Own Device” [BYOD]) showed strong evidence supporting equivalence between BYOD, paper, and a provisioned electronic device [16,17]. In the context of this study, data collection relative to treatment adherence, bleeding, bleeding treatment, HRQoL, and VAS was performed directly by the participants through a dedicated ePRO application downloaded and run on their devices [18]. In addition, participants were asked to wear the Fitbit Versa Health and Fitness Smartwatch (2018—Fitbit Inc. manufactured in China, designed in San Francisco, CA, USA—fitness tracker), a reliable and accurate system device to track PA [19,20] and sleep [21,22] to standardize these measurements.

### 2.3. Setting and Patients

This study was conducted in 18 investigational sites in Italy, which are highly representative of HA management in the country.

Subjects aged 12–50 years with severe (FVIII < 1%) or moderate (with FVIII of 1% to ≤2%, considered as “moderately severe”) congenital HA without current inhibitors or with previously tolerized inhibitors since at least 3 years and with at least 150 exposure days to FVIII were eligible if they were judged able to use the ePRO application and the fitness tracker. All participants and/or their caregivers should have signed informed consent before participating in this study.

Subjects with allergy or intolerance to any of the components of the device, hypersensitivity associated with monoclonal antibodies, clinically important risk or limiting diseases or conditions, obesity, ongoing or planned immune tolerance induction, or subjects participating in any interventional study were excluded.

### 2.4. Variables

The primary and secondary study objectives were evaluated overall and according to 3 age strata (i.e., 12–17 [adolescents], 18–30 [young adults], and 31–50 [adults] years).

The primary objective of this study was to describe PA over 12 months in subjects with HA without inhibitors in terms of duration and type of PA, distance covered, calories burned, body weight, heart rate, sleep data, active minutes, metabolic equivalents for tasks (METs), and step counts. All these data were collected through a fitness tracker and coded according to its software [23]. Time spent for PA was recorded by the tracker according to two different classifications: heart zone minutes (out-of-range, fat-burn, cardio, and peak minutes depending on the intensity of PA measured through the heart rate) and active zone minutes (sedentary, lightly, fairly, and very active minutes depending on the intensity of the PA measured through the heart zone minutes and other variables collected by the fitness tracker). Due to the configuration of the fitness tracker, the correspondence between the two classifications was not available as well as the total wearing time since the calculation of the resting time (out-of-range or sedentary minutes) was not directly derivable from the fitness tracker. The secondary objectives included the documentation of PA by type and intensity, and the description of the population in terms of adherence to WHO Guidelines for PA [24] disease severity, treatment regimen for HA, health status, joint health status, and BMI (kg/cm^2^). Adherence to WHO 2010 guidelines for the definition of PA was derived by using the active minutes recorded by the fitness tracker worn by the subjects during the study. A subject was defined as “adherent” to WHO guidelines according to the age and the number/intensity of active minutes spent per day/week. The incidence, treatment, and severity of bleeding, pain intensity, use of painkillers, and hospitalization/missed days at school/work related to the disease were also evaluated.

Among the exploratory objectives, the study aimed to evaluate the relationship between PA (type and intensity) and bleeding, and to observe participants for an additional 6 months if they switched to new therapies that became available during the study.

### 2.5. Data Sources

PA data were collected continuously using the fitness tracker and transferred to the study database. The compliance to the use of the fitness tracker was calculated, for each participant, as the ratio between the total number of valid days (i.e., a day in which the fitness tracker was worn for at least 10 h) and the number of days in the study.

HRQoL was evaluated using the European Quality of Life-5 Dimensions (EQ5D-5L) scale [25], completed by the subjects through the ePRO app at baseline and monthly. The HJHS (version 2.1) [26], which assesses joints most affected by bleeding (knees, ankles, and elbows), was completed at baseline, at six months, and at the end of the study.

A 100 mm VAS was used to assess pain intensity and was reported at baseline and monthly through the ePRO app.

Bleedings, including site and type of bleeding, (spontaneous, traumatic, or related to procedure/surgery), time of bleeding (date, start time, and end time), symptoms, and treatment, were recorded by the subject through the ePRO app [27] at time of bleeding or monthly.

Data on hospitalization and days away from school and/or work were collected monthly through the ePRO app, too. During the baseline visit, demographic data, medical history, data of PA, and medications taken in the four weeks before baseline were collected from the patient’s medical records; concomitant diseases and vital signs were evaluated at the baseline and follow-up visits. All data were transferred to the eCRF for evaluation by investigators.

### 2.6. Determination of Sample Size

Given the exploratory nature and the objectives of this study, a justification based on Italian epidemiologic data was considered appropriate. The planned sample size (approximately 150 subjects overall, 125 subjects with severe and 25 with moderate HA) was estimated to be representative of approximately 10–15% of Italian hemophilic subjects in the same age range and severity level planned for this study [23]. However, the sample was reduced to 107 due to the early interruption of enrollment related to the COVID-19 pandemic. This reduction did not cause any significant deviation from the planned precision of the estimates.

### 2.7. Patient and Public Involvement

This research was co-created with a hemophilia patient advocacy group representative who actively participated in setting research priorities, defining research questions and outcome measures, and providing input into the study design, conduct, data analysis, dissemination of results, and evaluation [18].

### 2.8. Statistical Considerations

Primary and secondary variables were analyzed via descriptive statistics. Continuous variables were summarized using mean, standard deviation, median, quartiles, and range (i.e., minimum and maximum). Categorical variables were summarized by using frequency and percentage distribution. The comparison among the age groups was performed with a Kruskal–Wallis test. The 95% confidence interval (CI) was also used to describe the main endpoints. All the analyses were performed using SAS 9.4. Data about bleeding, missed school/workdays, and hospitalization during the 24 weeks before baseline were collected verbally by the study physician during the baseline visits and reported on the patient chart, while the data during the observation period were systematically collected through the fitness tracker and the ePRO app.

### 2.9. Populations for Analysis

All participants who met the inclusion criteria were defined as the enrolled population (intention to treat—ITT). All the enrolled subjects with at least six valid months of PA evaluations through the fitness tracker who wore the fitness tracker for at least 10 h/day in at least half of the 6 months constituted the evaluable population (EVL), to warrant a strict statistical evaluation of the PA outcomes. All patients who switched to a new treatment for their HA constituted the switch population.

The results related to the PA outcome have been described only for the EVL population where all patients have at least 180 days of exposure to the fitness tracker, while all the other outcomes have been described for the ITT population.

The results related to the switch to a new treatment have been described in the switch population. An ad hoc analysis of the patients entered in the study from 1 March to 4 May 2020 [1st lockdown] and from 13 October to 31 December 2020 [2nd lockdown] was conducted during the study to evaluate the influence of the COVID-19 pandemic on the parameters collected in these patients.

## 3. Results

### 3.1. Participants’ Characteristics and Follow-Up

**Figure 1:**
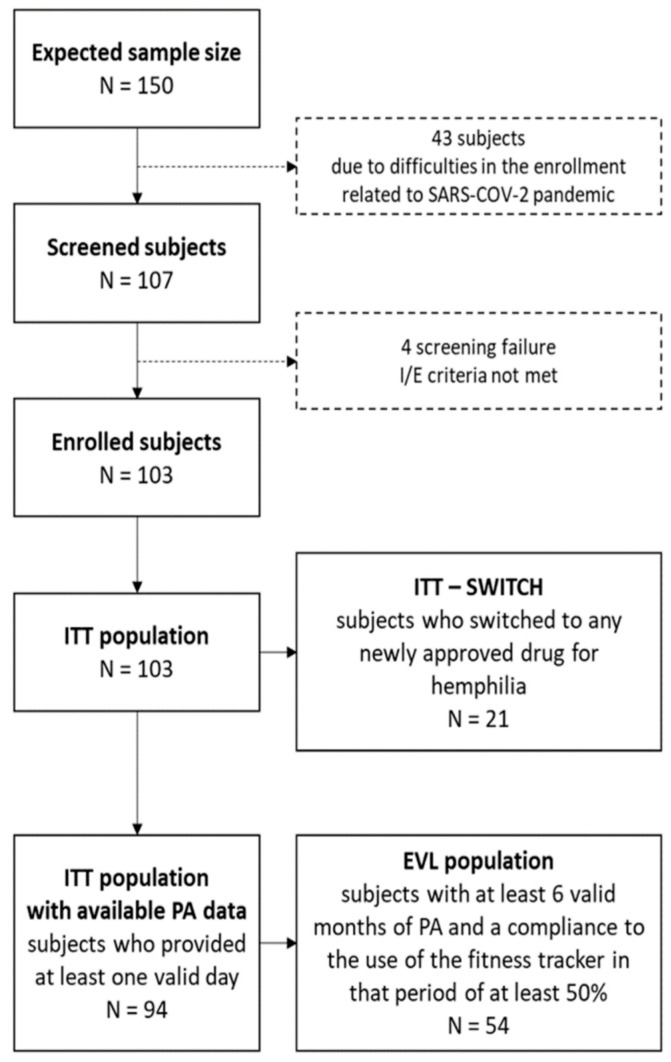
Patient flowchart and distribution by age group.

A total of 107 subjects were enrolled in the study; 103 of them were eligible according to the inclusion/exclusion criteria, and 54 of them constituted the EVL. Adolescents were the less-represented subgroup. A total of 15 subjects did not complete the 12-month observational period (10 because of the patient’s inability or unwillingness to comply with protocol requirements or non-compliance despite appropriate education measures taken by the clinical site, and 5 withdrew their informed consent) (Figure 1).

During the study, 21 (20.4%) subjects switched to a newly approved drug for HA without inhibitors.

At baseline, all the participants were males aged between 12 and 50 years with a mean (SD) BMI of 23.3 (3.6) kg/m^2^ and reported doing physical exercise in their spare time (Table 1).

About 90% of participants had a confirmed diagnosis of severe HA (Table 1); none of the included subjects had a history of thrombosis or anaphylaxis.

All participants (ITT) received treatment for HA at baseline; 88 (86.3%) were using a blood coagulation factor, and 14 (13.7%) a monoclonal bispecific antibody (emicizumab).

During the observational period, over the ITT population, 102 subjects received at least 1 prophylaxis therapy: 84.3% of subjects (n = 86) were on prophylaxis with blood coagulation factors (32.4% octocog alfa [n = 33]; 21.6% efmoroctocog alfa [n = 22]; 7.8% turoctocog alfa [n = 8]; 5.9% moroctocog alfa [n = 6]; 4.9% rurioctocog alfa pegol [n = 5]; 3.9% lonoctocog alfa [n = 4]; 2.9% damoctocog alfa pegol [n = 3]; 2.0% simoctocog alfa [n = 2]; 2.0% turoctocog alfa pegol [n = 2]; and 1.0% factor VIII no better specified [n = 1]); 15.7% (n = 16) of subjects were on prophylaxis with no-replacement therapy (emicizumab); and 1 subject received an on-demand therapy.

### 3.2. Compliance with the Fitness Tracker

A total of 54 subjects (52.4%) were using their fitness tracker for at least 10 h in 50% of their days for at least 6 months in the study; thus, less than half of the subjects included in the ITT population had compliance of at least 50% during the study. The use of the fitness tracker increased with participants’ age (Table 2). The median exposure to the use of the fitness tracker was 333 (143; 371) days.

A total of 54 participants (52.4%) included in the study were included in the EVL population. The exposure to the fitness tracker was above 180 days as required according to the study protocol, and the median compliance in the observation period was 73% (IQR 63.4; 92.4).

### 3.3. Primary Endpoint: Physical Activity

**Figure 2:**
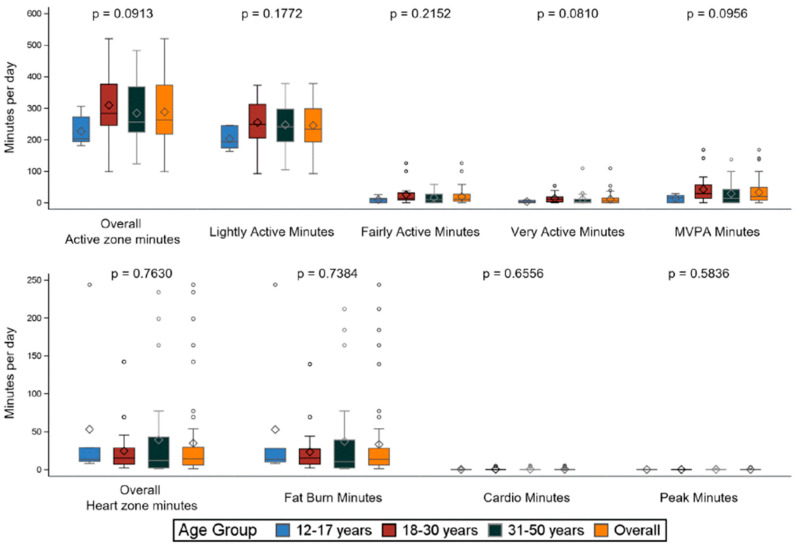
Physical activity: comparison by age group—EVL population. ° are outliers. Legend—EVL population includes ITT subjects with an exposure of at least 6 months and compliance of at least 50% within the period’s first–last valid day. MVPA = moderate to vigorous physical activity. *p* values are calculated by comparing age groups with a Kruskal–Wallis test.

The results on physical activity are shown for the EVL population. The 6 adolescents were in the active zone for a median (Q1–Q3) of 203.0 (194.0–272.0) min per day, the 22 young adults for 283.5 (245.5; 376.0) min, and the 26 adults for 255.5 (224.0; 368.0) minutes (*p* = 0.0913). Most of the active minutes were light (adolescents 193.3 [174.0;244.0], young adults 248.5 [205.5; 312.0], and adults 240.3 [194.0;297.0], *p* = 0.1772). Data collected as heart zone minutes show similar results with a great part of the time spent in the fat-burn heart zone (median [Q1–Q3] 13.5 [6.0–28.0] min) during the overall time registered in the overall heart zone (median 14.0 [6.0–29.5] min) (Figure 2).

**Figure 3:**
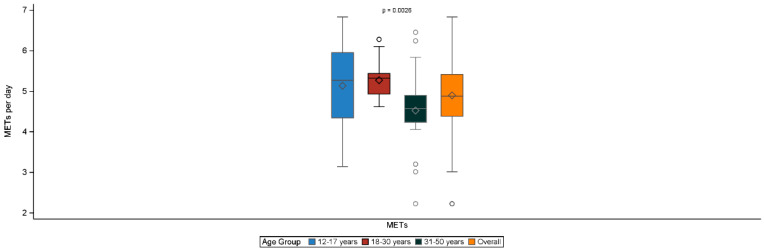
Metabolic equivalent of task (MET)—EVL population. ° are outliers. Legend—EVL population includes ITT subjects with an exposure of at least 6 months and compliance of at least 50% within the period’s first–last valid day. *p* values are calculated by comparing age groups with a Kruskal–Wallis test.

The median (Q1–Q3) METs (adolescents 5.3 [4.3–6.0] kcal/kg/hour, young adults 5.3 [4.9–5.4] kcal/kg/hour, and adults 4.6 [4.2–4.9] kcal/kg/hour, *p* = 0.0026) resulted statistically differently in the various age groups (Figure 3), while the number of steps per day (adolescents 4648 [3775–6411], young adults 8385 [6718–10,287], and adults 7274 [5088–10,532] steps, *p* = 0.0649) resulted similarly between the various age groups.

### 3.4. Secondary Endpoints

**Figure 4:**
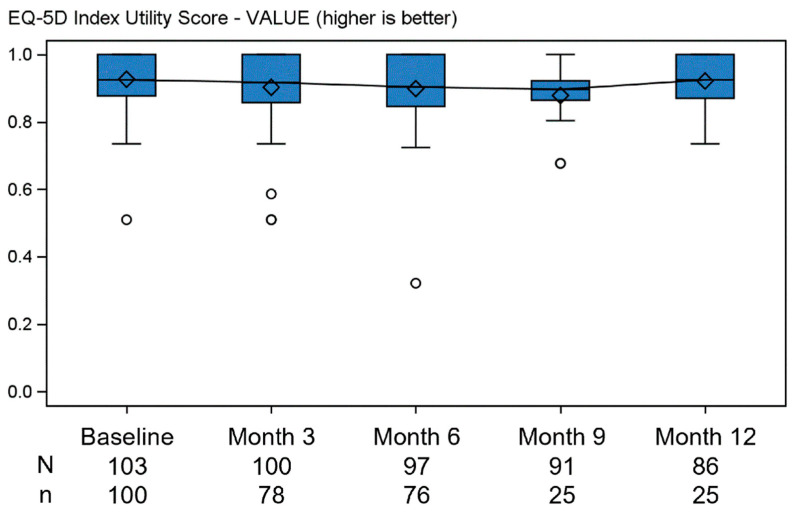
EQ-5D utility score by visit—ITT population. ° are outliers. Legend—N is the number of subjects in the ITT population at each time point; n is the number of subjects in the ITT population for which the value is collected at each time point. The line joins the medians. The diamonds represent the mean.

**Figure 5:**
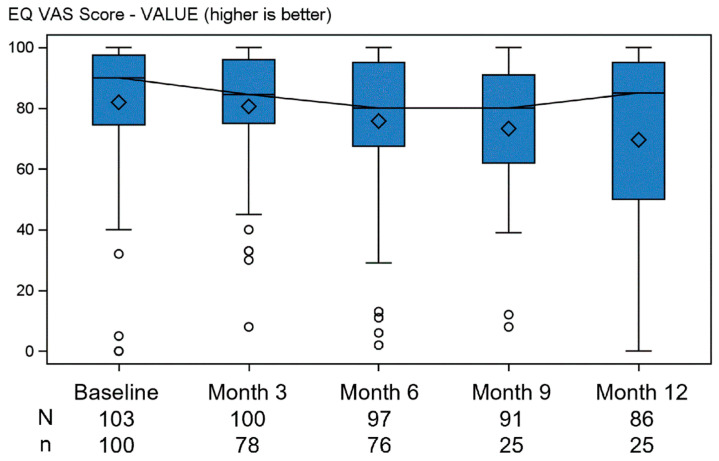
EQ-EQ VAS Score by visit—ITT population. ° are outliers. Legend—N is the number of subjects in the ITT population at each time point; n is the number of subjects in the ITT population for which the value is collected at each time point. The line joins the medians. The diamonds represent the mean.

Health-related quality of life and health status, measured through the EQ-5D utility score (Figure 4), had median (Q1–Q3) values of 0.92 (0.88–1.0) at baseline and 0.92 (0.87–1.0) at 12 months; the EQ VAS score (Figure 5) had median (Q1–Q3) values of 90.0 (74.5–97.5) at baseline and 85.0 (50.0–95.0) at 12 months.

The frequency of answers to each domain of the EQ-5D did not show any notable variation at the various time points (Appendix A).

The median (Q1–Q3) total score of the HJHS, evaluated on the whole ITT population, varied from 4.0 (0.0–15.0) at baseline to 5.0 (0.0–15.5) at the end of the study (Appendix B). The corresponding global gait score did not show any variation (baseline and 12 months median (Q1–Q3) 0.0 (0.0–2.0).

Pain intensity, measured through the VAS for pain, varied from a median (Q1–Q3) value of 4.0 (0.0–20.0) at baseline to 11.5 (0.5–31.5) at 12 months.

During the study, 13 (12.6%) subjects of the ITT and 6 (11.1%) subjects of the EVL population were hospitalized at least once.

The subjects were away from school for a mean of 3 (8.3) days/year, while parents/caregivers were away from work for a mean of 2.2 (8.5) days a year.

#### 3.4.1. Bleeding

In the ITT population, 45 (43.7%) subjects experienced at least one bleeding in the observation period; a total of 129 episodes, corresponding to an overall annual rate (95% CI) of 1.351 (1.127; 1.606) bleeding/year, were observed, mainly at joints (n = 83) and having traumatic origin (n = 81) (Table 3). Most of the bleedings (90, 69.8%) were treated by administering factor VIII via the intravenous route; none of them required a transfusion and 4 (3.1%) required additional hemostatic therapy. The most common symptom associated with the bleed reported by subjects was pain.

#### 3.4.2. Adherence to WHO Guidelines

**Figure 6:**
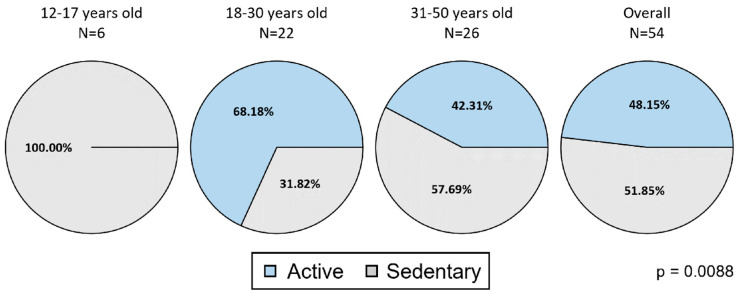
Adherence to the WHO guidelines for physical activity—EVL population. Legend—EVL population includes ITT subjects with an exposure of at least 6 months and compliance of at least 50% within the period’s first–last valid day. A subject was defined as “adherent” to WHO guidelines according to the following algorithm: 12–17 years old: MVPA (moderate to vigorous physical activity) minutes per day ≥ 60 min; 18–50 years old: fairly active (moderate) minutes per week ≥ 150 min OR very active (vigorous) minutes per week ≥ 75 min OR an equivalent combination of fairly and very active minutes (i.e., considering 1 fairly active minute as 0.5 very active minutes). *p* values are calculated by comparing age groups with a Kruskal–Wallis test.

According to the WHO criteria, there was a statistically significant difference in the frequency of PA among the age groups (*p* = 0.0088): all the adolescents included in the EVL showed sedentary behavior while the frequency of active subjects was higher in young adults of the EVL population (15/22, 68.2%) than in adults (11/26, 42.3%) (Figure 6). Similar results can be observed in the ITT population (*p* = 0.0005).

Most of the patients in the EVL population with sedentary behavior (28–51.9%) had severe HA at enrollment. In the EVL population, the rate of subjects who had at least one hospitalization during the observational period was higher in subjects with sedentary behavior (5 patients, 17.9%) than in subjects with active behavior (1 patient, 3.8%) (Table 4).

The median (Q1–Q3) yearly rate of bleeding in the EVL population was numerically lower in subjects with active behavior (1.384 [0.974–1.907] bleeding/year) than in subjects with sedentary behavior (1.945 [1.481–2.509] bleeding/year). A numerically lower proportion of active subjects experienced one or more bleeding(s) (13–50.0%) than sedentary ones (16–57.1%) (Table 4).

Health-related quality of life did not show any significant variation during the study in the EVL population, while the mean (SD) EQ-5D VAS score decreased (i.e., worsened) from baseline to any post-baseline time point in subjects with sedentary behavior and did not substantially change in subjects with active behavior (except for a mean decrease at month 9) (Table 5).

In the EVL population, the mean (SD) HJHS total score increased (i.e., worsened) from baseline to month 12 in both subjects with sedentary and active behavior, with more marked mean increases in subjects with sedentary behavior at both time points (Table 5). The mean (SD) global gait score did not substantially change from baseline to month 6 and month 12.

The mean (SD) VAS score for pain had a similar trend (i.e., worsening) in both active and sedentary subjects (Table 5).

#### 3.4.3. Switch to a New Therapy

Twenty-one subjects switched to a newly approved drug for HA without inhibitors: 8 [38.0%] to a non-replacement therapy with emicizumab, 13 [62%] to a replacement therapy with turoctocog alfa pegol [n = 7–33%], or rurioctocog alfa pegol [n = 3–14%], or damoctocog alfa pegol [n = 3–14%]. These subjects were observed for an additional 6 months after the switch date. Their clinical and demographic characteristics were similar to those of the main study sample (Table 6).

Fourteen (66.7%) of the 21 subjects who switched their treatment showed sedentary behavior before switching. Subjects with active behavior increased from six (28.6%) before the switch to eight (38.1%) after switching to a newly approved drug for HA. Six (28.6%) subjects experienced one or more bleeds before the switch and three (14.3%) after switching. The health-related quality of life, the joint health state, BMI, and pain intensity did not substantially change after switching (Table 7).

#### 3.4.4. Impact of COVID-19 on the Study Results

The ad hoc analysis to evaluate the impact of the COVID-19 pandemic on the study endpoints was performed during the study. The three periods analyzed were defined as (i) the first lockdown period with government restrictions (from 1 March 2020 to 4 May 2020); (ii) the second lockdown period with government restrictions (from 13 October 2020 to 31 December 2020); and (iii) no lockdown period without or fewer restrictions. A total of 8 patients (1 adolescent, 2 young adults, 5 adults) were included in the study at the time of the first lockdown and 66 patients (12 adolescents, 28 young adults, 26 adults) were enrolled at the time of the second lockdown period to evaluate the effect of the pandemic on the patient’s habits.

The adherence to the WHO recommendations observed in patients included in the COVID-19 periods analysis was consistent with the one observed in the overall ITT and EVL populations.

The mean (SD) adherence to the treatment regimen for HA was 75.0 (31.2%) during the first lockdown period, 69.7 (25.1%) during the second lockdown period, and 54.8 (28.8) out of the lockdown period. A total of 3 (37.5%) patients had one bleeding during the first lockdown period, while 13 (19.7%) had one or more bleeding (mean [SD]: 2.0 [1.2]) during the second lockdown period. Out of the lockdown periods, 39 (37.9%) patients had one or more bleeding (mean [SD]: 2.6 [2.6]).

The analyses of the physical activity, quality of life, health status, and pain did not show any variation in any of the examined parameters during the lockdown when compared with the other study periods (Appendix C).

## 4. Discussion

In this study, 103 subjects, mostly with severe HA without inhibitors, underwent 12-month monitoring of their daily PA using a commercial fitness tracker. Our findings revealed that subjects perform less PA than recommended by the WHO guidelines, even if possibly motivated by participating in a cohort study.

No clinically significant variations have been observed in the subject’s vital signs, quality of life, sleep, pain, or joint status during the 12 months of the observation in any of the study groups and overall. The fitness tracker was used less than recommended, especially by adolescents. The analysis of the different groups showed that adolescents have sedentary behavior, even if 75% of them report regularly exercising in their free time at baseline, while the PA levels in the other age groups confirm that most of the subjects with HA do not engage in enough PA according to the WHO recommendation [24]. This finding is consistent with that of Norwegian teenagers and young adults with HA compared to their general population peers [9]. Bleeding was observed in about half of the enrolled subjects, which is lower than observed in other recent studies but still present [28,29,30]. Consistently with other studies [9,10,31,32], our results show that most of the subjects have scarce PA both as time spent and intensity. Despite the limited PA, subjects reported bleeding, especially observed in the population with sedentary conditions. This could suggest that other causes than PA, for example, treatment adherence and type of treatment, might affect the number, frequency, and severity of bleeding [29,32,33,34]. These outcomes might be particularly relevant considering the stringent monitoring of bleeding and PA being more accurate than in other studies and belonging to data systematically collected for 12 months in the real-world environment [35].

There are strengths and weaknesses of the study to be noted. This is the first study using a commercially available fitness tracker to evaluate the PA of subjects with HA for at least one year. The use of the same fitness tracker by all the participants, together with the data collection using the ePRO app, permitted the “on-stage” collection of consistent and comprehensive data by the subjects, and a systematic and deeply controlled approach for the data collection.

The study was conducted during the COVID-19 pandemic, which hugely affected the lives of the subjects and their care [36]. The analysis of the main study variables during the COVID-19 pandemic, by lockdown period, although limited by variability in the number of evaluable patients in each period, contributes to a better understanding of the impact of the strict social and activity limitations adopted by the Italian government during the pandemic. Differently from other studies depicting a decrease in PA among adolescents and young adults during the first COVID-19 lockdown in the Netherlands [37], Norway [9], and France [38], our study did not reveal any impact on the PA recorded both in the first and second lockdown. It is important to consider that the observation period in our ad hoc analysis was longer, all data related to PA were monitored (not only sports), and the data collection solution was more accurate despite the e-survey. On the other hand, our results confirm those from other studies revealing that patients’ well-being and lifestyle, treatment adherence, bleeding frequency, pain, and painkiller use were not affected by the pandemic [9,36,38].

The comparison between the data collected during the pandemic period with most restrictions with data collected during the period with fewer restrictions seems not to show any significant difference in our study, either. Anyway, we cannot completely ignore the COVID-19 influence while interpreting the results of the study.

As about half of the enrolled subjects did not use the fitness tracker for at least 10 h per day, the EVL population has been analyzed to warrant a reliable evaluation of the PA.

Due to the observational nature of this study, the small sample of non-severe subjects, the subjects treated with the different drugs, the variables affecting patient outcomes, and their mutual interactions were not studied.

Approximately two-thirds of subjects overall, and all adolescents, showed sedentary behavior according to WHO guidelines. The results from this study confirm the need for tailored and appropriate PA, especially for younger subjects with HA [4], since the study results seem to suggest that the intensity of PA does not affect the risk of bleeding. Any effort to continue to investigate the relationship between PA and bleeding to promote more adherence to the WHO guidelines [39] is looked forward to as the next step for improving the management of these subjects.

Our results show the feasibility of studying PA in subjects with HA without inhibitors using a commercially available fitness tracker. The algorithm encoding the movements recorded by the fitness tracker is not publicly available and has been updated by the producer, without advising the users, during the study. This highlights the need for a stricter collaboration between fitness tracker producers and researchers in future studies to understand better the clinical meaning of the values collected through the fitness tracker. A study on a broader sample of participants should look forward to identifying the factors predicting the influence of PA on well-controlled subjects with HA, on the clinical status and daily life of persons with HA and their caregivers.

## 5. Conclusions

The use of a fitness tracker and an ePRO app allows the systematic recording of data on the daily activity, QoL, bleeding, and adherence to therapy in subjects with HA without inhibitors in their daily life.

Approximately two-thirds of subjects overall, and all subjects in the adolescent cohort (aged 12–17 years), had sedentary behavior based on adherence to WHO Guidelines for the definition of physical activity.

Bleedings (spontaneous or traumatic) were reported in 43.7% of subjects and were reported in a higher proportion of subjects with sedentary behavior than in subjects with active behavior. The physical activity in the 2 days preceding the bleeding was comparable to the one observed in the overall observational period.

These data suggest that the intensity of PA is not directly linked with an increased risk of bleeding. However, further research on a larger sample of patients is needed to confirm the relationship between bleeding and physical activity.

Adherence to the treatment was quite poor, needing great insight from the physicians to make the subjects aware of its crucial importance.

A sedentary condition seems to be associated with a worse pain status, a higher risk of hospitalization, and a worse joint status compared to active subjects.

The results from this study show that persons with HA without inhibitors have lower-than-expected physical activity, that bleeding, pain, and adherence to the treatment regimen are still not fully controlled, and that they were not significantly affected by the COVID-19 restrictions during the observed period.

The analysis of changes following the switch could suggest that switching to any newly approved drug may be associated with increased adherence to WHO Guidelines for PA and a decrease in the risk of bleeding. Due to the small sample size, further analysis should be conducted to better investigate the impact that the different types of therapies might have on these outcomes.

## Figures and Tables

**Table 1:** Demographic and clinical characteristics at baseline.

	ITT Population				EVL Population			
	12–17 Years (N = 17)	18–30 Years (N = 46)	31–50 Years (N = 40)	Overall (N = 103)	12–17 Years (N = 6)	18–30 years (N = 22)	31–50 Years (N = 26)	Overall (N = 54)
Age (years)								
Mean (SD)	14.1 (1.6)	24.1 (3.8)	39.1 (5.8)	28.3 (10.4)	14.5 (2.2)	24.6 (3.8)	39.2 (5.9)	30.5 (10.1)
Min; Max	12; 17	18; 30	31; 50	12; 50	12; 17	19; 30	31; 50	12; 50
BMI (kg/m^2^)								
Mean (SD)	20.1 (3.6)	23.7 (3.1)	24.4 (3.4)	23.3 (3.6)	19.7 (4.0)	23.5 (3.3)	24.3 (3.3)	23.5 (3.6)
Min; Max	16.1; 27.3	18.7; 29.6	17.0; 29.9	16.1; 29.9	16.1; 25.4	18.7; 29.6	17.5; 29.9	16.1; 29.9
Time from diagnosis (years)								
Mean (SD)	13.3 (2.0)	22.6 (4.6)	36.0 (9.2)	26.3 (10.6)	13.5 (2.6)	23.5 (4.4)	35.8 (10.7)	28.3 (11.1)
Min; Max	10; 16	12; 30	0; 50	0; 50	10; 16	15; 30	0; 50	0; 50
Severity								
(n (%))								
Moderate	2 (11.8)	3 (6.5)	1 (2.5)	6 (5.8)	0	3 (13.6)	1 (3.8)	4 (7.4)
Severe	15 (88.2)	43 (93.5)	39 (97.5)	97 (94.2)	6 (100.0)	19 (86.4)	25 (96.2)	50 (92.6)
History of positive inhibitors against FVIII (n (%))	2 (11.8)	5 (10.9)	3 (7.5)	10 (9.7)	1 (16.7)	2 (9.1)	2 (7.7)	5 (9.3)
Time since the inhibitor was eradicated (years)								
Mean (SD)	10.0 (1.4)	17.6 (6.5)	25.7 (0.6)	18.5 (7.3)	11.0	19.5 (12.0)	26.0 (0.0)	20.4 (8.6)
Min; Max	9; 11	11; 28	25; 26	9; 28	11; 11	11; 28	26; 26	11; 28
Bleedings during 24 weeks before baseline (n (%))								
0	13 (76.5)	35 (76.1)	25 (62.5)	73 (70.9)	4 (66.7)	19 (86.4)	20 (76.9)	43 (79.6)
1/2	4 (23.5)	6 (13.0)	6 (15.0)	16 (15.5)	2 (33.3)	2 (9.1)	4 (15.4)	8 (14.8)
3/4	0	3 (6.5)	4 (10.0)	7 (6.8)	0	1 (4.5)	1 (3.8)	2 (3.7)
5/6	0	0	0	0	0	0	0	0
>6	0	0	5 (12.5)	5 (4.9)	0	0	1 (3.8)	1 (1.9)
Unknown	0	2 (4.3)	0	2 (1.9)	0	0	0	0
Subjects who regularly exercise in their free time (n (%))	13 (76.5)	33 (71.7)	23 (57.5)	69 (67.0)	5 (83.3)	16 (72.7)	12 (46.2)	33 (61.1)

Legend—ITT population: intention-to-treat population (all the enrolled subjects); EVL population includes ITT subjects with an exposure of at least 6 months and compliance of at least 50% within the period first–last valid day; SD: standard deviation; BMI: body mass index.

**Table 2:** Compliance during the observation period.

	ITT Population				EVL Population [3]			
	12–17 Years (N = 17)	18–30 Years (N = 46)	31–50 Years (N = 40)	Overall (N = 103)	12–17 Years (N = 6)	18–30 Years (N = 22)	31–50 Years (N = 26)	Overall (N = 54)
Exposure (days)								
Median	327	323	343	333	354	372	361	362
Q1; Q3	141; 362	136; 373	216; 368	143; 371	327; 363	348; 378	342; 372	342; 376
Compliance during the observational period								
Median	41.4	44.4	54.7	46.2	60.4	73.7	76.5	73.0
Q1; Q3	16.7; 48.5	12.9; 73.4	22.3; 82.2	13.7; 73.4	46.2; 71.0	54.5; 91.1	58.3; 90.8	54.5; 85.9
Compliance during the period defined by the exposure								
Median	43.4	57.4	72.7	58.6	65.5	77.8	81.0	77.4
Q1; Q3	23.2; 59.8	34.8; 85.9	43.4; 84.6	33.9; 82.9	55.1; 74.3	63.4; 92.8	69.1; 92.4	63.4; 92.4

Legend—The exposure to the use of the fitness tracker is derived on a per-patient basis as: date of the last valid day–date of the first valid day + 1, where a valid day is defined as a day in which the fitness tracker is worn for at least 10 h. The fitness tracker’s compliance is derived on a per-patient basis as the ratio between the total number of valid days during the observational period and the length of the observational period × 100 or the exposure to the fitness tracker × 100. The EVL population includes ITT subjects with an exposure of at least 6 months and compliance of at least 50% within the period first–last valid day.

**Table 3:** Bleedings during the observation period.

	12–17 Years (N = 17)	18–30 Years (N = 46)	31–50 Years (N = 40)	Overall (N = 103)
Total number of bleedings per patient				
Number (%) of subjects with bleeding	6 (35.3%)	21 (45.7%)	18 (45.0%)	45 (43.7%)
Median number of bleedings	1.5	2.0	2.5	2.0
Q1; Q3	1; 2	1; 3	2; 5	1; 3
Spontaneous bleeds				
Number (%) of subjects with bleeding	2 (11.8%)	10 (21.7%)	7 (17.5%)	19 (18.4%)
Median number of bleedings	1.5	1.0	2.0	2.0
Q1; Q3	1; 2	1; 2	2; 5	1; 2
Traumatic bleeds				
Number (%) of subjects with bleeding	3 (17.6%)	18 (39.1%)	14 (35.0%)	35 (34.0%)
Median number of bleedings	1.0	2.0	2.0	2.0
Q1; Q3	1; 4	1; 3	1; 4	1; 3
Bleeds related to procedures/surgeries				
Number (%) of subjects with bleeding	0	0	1 (2.5%)	1 (1.0%)
Median number of bleedings	0	0	1.0	1.0
Q1; Q3			1; 1	1; 1

**Table 4:** Disease severity, hospitalizations, and patients with bleeding by physical activity—EVL population.

		Subjects with Active Behavior N = 26	Subjects with Sedentary Behavior N = 28	Overall N = 54
Hemophilia A severity	Moderate	3 (75.0%)	1 (25.0%)	4 (7.4%)
	Severe	23 (46.0%)	27 (54.0%)	50 (92.6%)
Hospitalizations	No hospitalizations	25 (96.2%)	23 (82.1%)	48 (88.9%)
	At least one hospitalization	1 (3.8%)	5 (17.9%)	6 (11.1%)
Patients with bleeding	No bleeds	13 (50.0%)	12 (42.9%)	25 (46.3%)
	1 or more bleeds	13 (50.0%)	16 (57.1%)	29 (53.7%)
Total number of bleedings		59	37	96
Bleeding by site				
Urinary, feces, vomit	No bleeds	26 (100.0%)	24 (85.7%)	50 (92.6%)
	1 or more bleeds	0	4 (14.3%)	4 (7.4%)
Joint	No bleeds	16 (61.5%)	18 (64.3%)	34 (63.0%)
	1 or more bleeds	10 (38.5%)	10 (35.7%)	20 (37.0%)
Muscles	No bleeds	21 (80.8%)	25 (89.3%)	46 (85.2%)
	1 or more bleeds	5 (19.2%)	3 (10.7%)	8 (14.8%)
Other	No bleeds	22 (84.6%)	21 (75.0%)	43 (79.6%)
	1 or more bleeds	4 (15.4%)	7 (25.0%)	11 (20.4%)
Spontaneous	No bleeds	17 (65.4%)	22 (78.6%)	39 (72.2%)
	1 or more bleeds	9 (34.6%)	6 (21.4%)	15 (27.8%)
Traumatic	No bleeds	15 (57.7%)	19 (67.9%)	34 (63.0%)
	1 or more bleeds	11 (42.3%)	9 (32.1%)	20 (37.0%)
Related to procedures/surgeries	No bleeds	26 (100.0%)	27 (96.4%)	53 (98.1%)
	1 or more bleeds	0	1 (3.6%)	1 (1.9%)

EVL population includes ITT subjects with an exposure of at least 6 months and compliance of at least 50% within the period’s first–last valid day. A subject was defined as “adherent” to WHO guidelines according to the following algorithm: 12–17 years old: MVPA (moderate to vigorous physical activity) minutes per day ≥ 60 min; 18–50 years old: fairly active (moderate) minutes per week ≥ 150 min OR very active (vigorous) minutes per week ≥ 75 min OR an equivalent combination of fairly and very active minutes (i.e., considering 1 fairly active minute as 0.5 very active minutes). An adherent subject is defined as a subject with an “active” behavior, and a non-adherent subject is defined as a subject with a sedentary behavior.

**Table 5:** Quality of life and pain by physical activity—EVL population.

		Active				Sedentary			
		Baseline	Value at 3 Months	Value at 6 Months	Value at 12 Months	Baseline	Value at 3 Months	Value at 6 Months	Value at 12 Months
EQ5D5L-index score	n	25	23	24	22 ^	28	25	24	22 ^
	Mean (SD)	0.93 (0.07)	0.90 (0.09)	0.90 (0.08)	0.89 (0.08)	0.90 (0.10)	0.89 (0.11)	0.87 (0.14)	0.89 (0.10)
EQ5D-VAS *	n	25	23	24	22 ^	28	25	24	22 ^
	Mean (SD)	78.1 (27.6)	81.8 (17.3)	76.1 (20.9)	66.7 (29.3)	79.9 (17.9)	76.9 (19.8)	73.1 (24.3)	81.7 (16.2)
HJHS *	n	26	NA	6	14	28	NA	6	18
	Mean (SD)	12.1(14.7)		8.7 (5.4)	15.0 (16.6)	11.8 (17.2)		29.3 (34.9)	17.3 (25.3)
VAS-PAIN *	n	25	23	24	22 ^	28	25	23	23 ^
	Mean (SD)	14.12 (22.57)	17.17 (21.63)	20.67 (23.54)	21.09 (20.58)	17.86 (23.25)	22.80 (20.40)	24.09 (25.34)	20.72 (24.25)

^ The values were collected at 11 months because of the small number of subjects who completed the questionnaires at 12 months. * An increase in the score means worsening of the symptoms. Legend—EQ5D: European Quality of Life-5 Dimensions; HA: hemophilia A; HJHS: joint health status; VAS: visual analogue scale. EVL population includes ITT subjects with an exposure of at least 6 months and compliance of at least 50% within the period’s first–last valid day. A subject was defined as “adherent” to WHO guidelines according to the following algorithm: 12–17 years old: MVPA (moderate to vigorous physical activity) minutes per day ≥ 60 min; 18–50 years old: fairly active (moderate) minutes per week ≥ 150 min OR very active (vigorous) minutes per week ≥ 75 min OR an equivalent combination of fairly and very active minutes (i.e., considering 1 fairly active minute as 0.5 very active minutes).

**Table 6:** Demographic and clinical characteristics of the subjects switched to a newly approved drug for hemophilia A without inhibitors during the study.

	12–17 Years (N = 3)	18–30 Years (N = 12)	31–50 Years (N = 6)	Overall (N = 21)
Age (years)				
Mean (SD)	14.0 (1.7)	24.6 (4.6)	34.7 (3.4)	25.9 (7.8)
Min; Max	12; 15	18; 30	31; 40	12; 40
BMI (kg/m^2^)				
Mean (SD)	21.4 (3.9)	23.8 (3.5)	25.0 (2.4)	23.8 (3.3)
Min; Max	16.9; 23.8	18.7; 29.6	20.6; 27.5	16.9; 29.6
Time from diagnosis (years)				
Mean (SD)	13.7 (2.3)	22.5 (6.1)	33.7 (3.6)	24.4 (8.3)
Min; Max	11; 15	12; 30	30; 39	11; 39
Severity (n (%))				
Moderate	0	2 (16.7)	0	2 (9.5)
Severe	3 (100)	10 (83.3)	6 (100)	19 (90.5)
History of positive inhibitors against FVIII (n (%))	0	2 (16.7)	2 (33.3)	4 (19.0)
Bleedings during 24 weeks before baseline (n (%))				
0	3 (100)	11 (91.7)	5 (83.3)	19 (90.5)
1/2	0	1 (98.3)	1 (16.7)	2 (9.5)

Legend—SD: standard deviation; BMI: body mass index.

**Table 7:** Clinical outcomes of the subjects switched to a newly approved drug for hemophilia A without inhibitors during the study.

	Before the Switch	After the Switch
BMI (kg/m^2^)		
N	21	11
Mean (SD)	23.9 (3.4)	24.1 (2.3)
Min; Max	16.9; 29.6	20.2; 28.0
Compliance with the use of the fitness tracker (%)		
N	21	19
Mean (SD)	64.8 (28.6)	54.2 (33.3)
Min; Max	0; 99	0; 97
Adherent to WHO guidelines		
N	20	18
n (%)	6 (30.0%)	8 (44.4%)
Overall active minutes per day		
N	20	18
Mean (SD)	266.8 (100.2)	297.8 (120.2)
Min; Max	98; 481	119; 547
Overall heart zone minutes per day		
N	20	18
Mean (SD)	46.6 (67.9)	32.0 (51.9)
Min; Max	2; 248	2; 219
Patients with 1 or more bleeding		
N	21	21
n (%)	6 (28.6)	3 (14.30)
EQ-5D Index Utility Score		
N	21	19
Mean (SD)	0.95 (0.06)	0.93 (0.06)
Min; Max	0.8; 1.0	0.8; 1.0
HJHS score		
N	21	15
Mean (SD)	6.5 (6.9)	7.8 (6.6)
Min; Max	0; 20	0; 20
VAS for pain score		
N	21	20
Mean (SD)	11.3 (14.0)	11.7 (14.1)
Min; Max	0; 50	0; 49

Legend—N: analyzed sample size; n: number of patients with the outcome; SD: standard deviation; BMI: body mass index; WHO: World Health Organization; EQ-5D: European Quality of Life-5 Dimensions; HJHS: joint health status; VAS: visual analogue scale.

**Figure A1:**
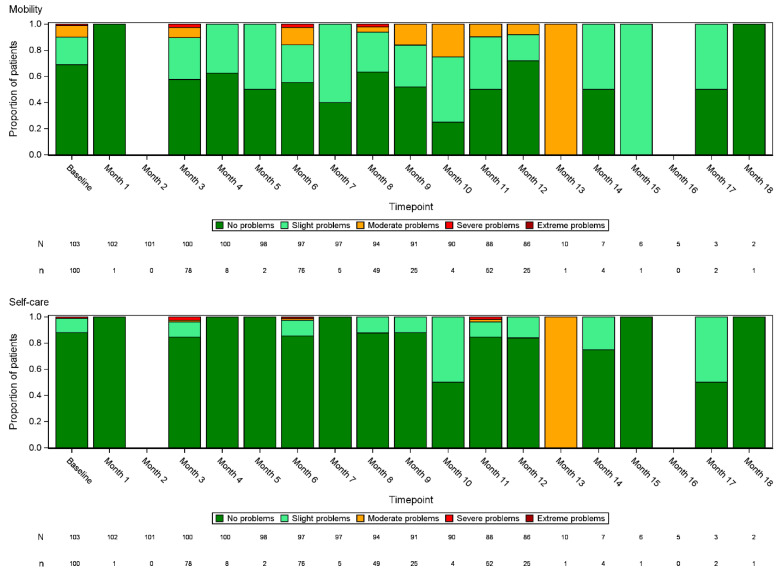

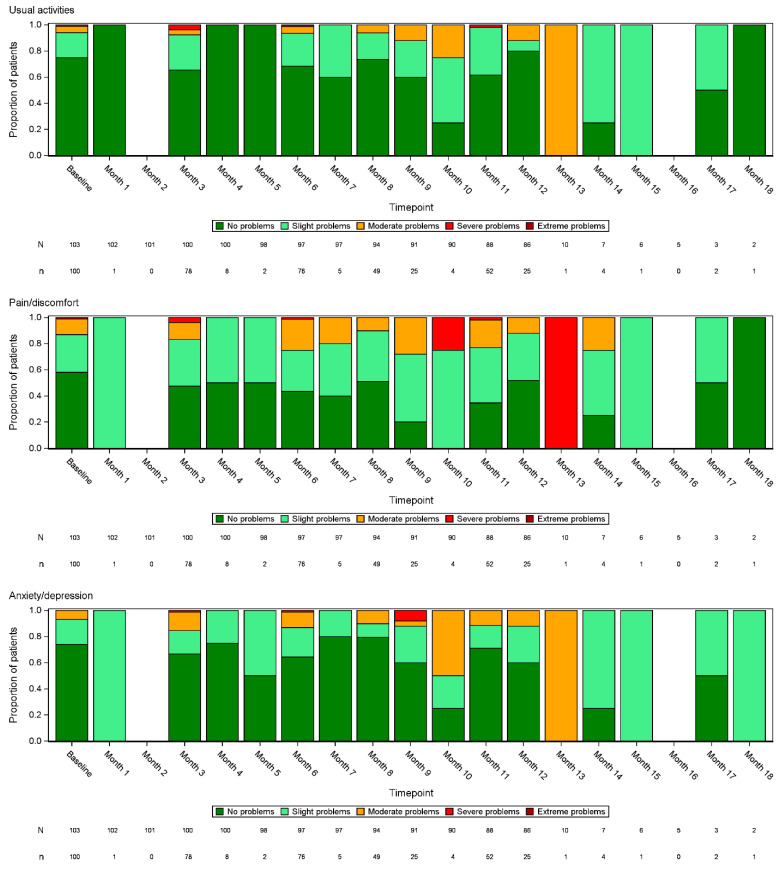
Legend—N is the number of subjects in the ITT population at each time point; n is the number of subjects in the ITT population for which the value is collected at each time point. Percentages are calculated on the number of subjects for which the value is collected at each time point.

**Figure A2:**
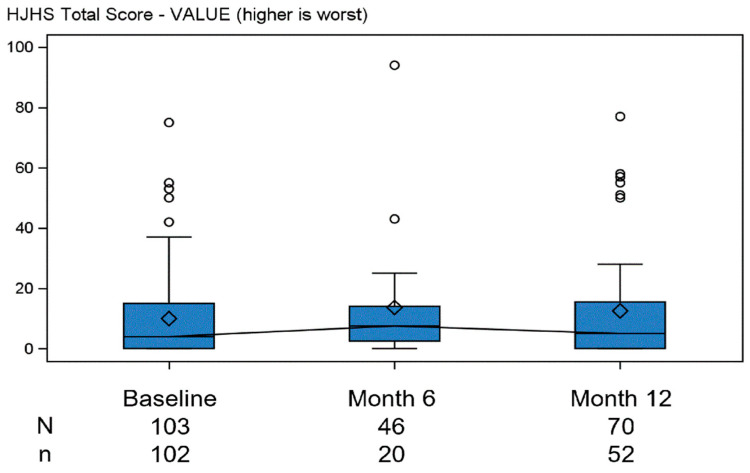
Legend—The joint health status was collected every 6 months. For subjects who switched to any newly approved drug for hemophilia during the observational period, data were also collected 6 months after the start of the administration of the new drug. N is the number of subjects in the ITT population who completed the specific observational visit; n is the number of subjects in the ITT population for which the value was collected.

**Figure A3:**
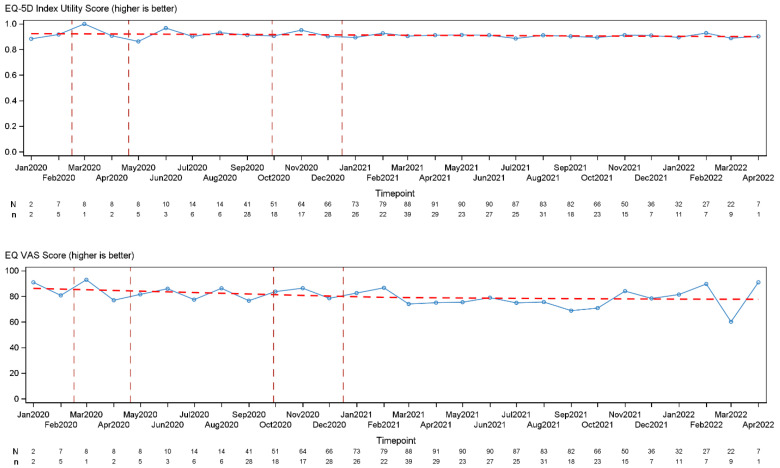

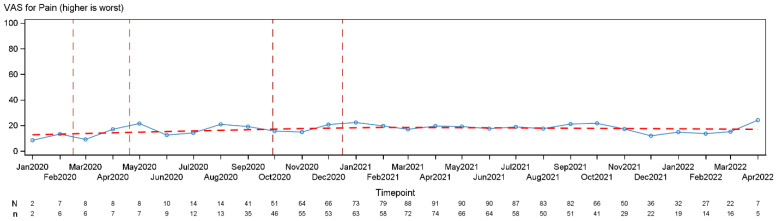
Legend—N is the number of subjects in each period; n is the number of subjects for which the value is collected at each time point. The vertical dashed lines define the two lockdown periods: the first period from 1 March 2020 to 4 May 2020, and the second period from 13 October 2020 to 31 December 2020. Data are summarized within each month by using the mean (blue markers in the graph). Data collected from 1 May 2020 to 4 May 2020 are summarized under April 2020. The blue line connects the means observed each month. The red line represents the estimated trend by using the Loess regression.

## Data Availability

The data that support the findings of this study are available on request from the corresponding author. For original data, please contact Sara Bendinelli (sara.bendinelli@roche.com). Individual participant data will not be shared.
